# Embodied simulation, body language, and symbolization: understanding somatic symptoms in psychoanalysis

**DOI:** 10.3389/fpsyg.2026.1642418

**Published:** 2026-02-02

**Authors:** Elena Markova, Gabriel Enache

**Affiliations:** 1 Moscow Institute of Psychoanalysis, Moscow, Russia; 2 Bucharest Association for Counseling and Psychoanalytic Psychotherapy, The International Neuropsychoanalysis Society Association, Romania

**Keywords:** body language, embodied simulation, psychoanalysis, somatic symptoms, symbolization

## Abstract

Classical psychoanalysis has traditionally focused on uncovering unconscious conflicts through language—the so-called `talking cure`. However, contemporary research underscores the importance of embodied simulation, body language, and their symbolic representation in understanding and addressing patient’s somatic symptoms. This occurs particularly when individuals seeking psychological help are unable to use spoken words to express their feelings, which is why communication between patient and therapist takes place at a pre-verbal level, within the space of “intercorporeality.” In this paper we explore the interplay between early development, mentalization, language acquisition and somatic disorders as failures of symbolization that manifest in bodily distress. In psychoanalytic psychotherapy these processes can be studied as transference-countertransference interactions. We describe the observed clinical phenomena in terms of intersubjective communication grounded in embodied simulation theory, showing how bodily symptoms emerge when mentalization fails and countertransference is enacted at a concrete, somatic level. For this, a clinical vignette illustrates how severe deficits in mentalization produce concrete somatic enactments and embodied countertransference in the therapist, suggesting a neurobiological substrate in pathological intersubjective resonance. We also compare a clinical vignette to human interaction with an AI as a vivid illustration of what happens when there is ‘nobody’ (or ‘no body’) as a human counterpart in communication. LLMs lack bodily resonance and therefore cannot substitute for the therapeutic processes that rely on embodied simulation. Thus, our paper describes phenomenological observations of interbody communication in therapy caused by symbolization failure, integrates several theoretical and experimental approaches (intersubjectivity, embodied simulation, interbrain synchronization) to explain it, points at further directions of scientific research and indicates potential dangers for therapists and patients using modern AI technologies.

## 1 Introduction

Nadelman (1990) noted that Freud’s article “Psychical Treatment” (Solms, 2024, vol. 7) could be seen as the basis for modern psychosomatic treatment. In that paper, Freud indeed lays a basis for a psychosomatic treatment of some physiological ills. However, the relation soma-mind or mind-soma seemed to divide the therapists and the research in later years: in some quarters, a focus on the somatic explanation even for psychic ailments; in others, a disregard of the somatic components of psychic disturbances. The history of psychosomatics is the history of the struggle of accepting that our minds and our bodies are related, that our body is not a separate object, that can be treated with pills and knives by specialist in white coates. As Freud made very clear in his “The question of lay analysis” (Solms, 2024, vol. 20) psychoanalysis should not be the reserve of medicine and the struggle to allow lays to practice psychoanalysis divided the psyconalytical organizations.

In the early years of the 20th century, many of Freud’s colleagues, students and followers initiated the research of bodily diseases ending up with the full blossom of the German psychosomatic school in the 1960s, after the WWII (Kutter, 1998). In this paradigm psychoanalysts were trying to explain all somatic diseases in terms of ‘psychic sources’ causing certain symptoms. In this pursue of a clear psychosomatic theory Franz Alexander (1948) famously suggested a full classification of bodily systems but later, with the emerging clinical evidence provided by other researchers, it became apparent that it is a too straightforward approach to psychosomatics (Marty, 1991).

Alongside with the German school of psychosomatics another branch of research was developing in France which is known as the Paris School of psychosomatics founded by Pierre Marty and Michel de M’Uzan. Although they described the emergence of physical symptoms as deficiencies in mentalization process, in fact the term ‘mentalization’ was initially introduced by Marty and de M’Uzan (1978). It was indeed a revolutionary approach rejecting Alexander’s specificity concept. Marty suggested that patients with psychosomatic disorders displayed a particular type of thinking which he called operative. He understood this as a failure of mental representation resulting in a very concrete way of interpreting the external world. The ideas of Marty have been further developed by Stora (2023) but in his research, notwithstanding the beauty and elegance of conceptual framing of clinical cases, it still remains unclear how mentalization function can be developed in patients with psychosomatic disorders.

The technical aspects of mentalization development have been rigorously grinded by Bateman and Fonagy and applied for the treatment of borderline personality disorder (Fonagy and Bateman, 2019). This method proved to be very helpful in this regard. Patients with severe attachment problems can develop this function within transference relationships. But these authors disregard psychosomatic issues in their treatment although such patients frequently experience bodily symptoms due to lack of mental container and ability or skill to process their emotional experiences.

Furthermore, we should not forget that mentalization is tightly linked to language since language is the main tool for the development of this psychical function. Psychoanalysis being a ‘talking cure’ helps patients learn how to use language to develop their personal self-narrative. Various psychoanalysts have been researching the aspect of language application in psychotherapy (Meltzer, 1984; Ogden, 1997) with Jacques Lacan (1998) among the most famous names in this regard, and Thomas Paul Bonfiglio (2023) the most recent one. And here again, we can observe language becoming the main focus while the body and somatic disorders seem to be overlooked.

Finally, since we are talking about somatic diseases it’s worth mentioning here that current research in somatic therapies due to medical traditions usually treats patients objectively, without taking into consideration the subjective aspects of the disease (Sánchez, 2023). Moreover, very few medical specialists practice holistic approach to the human organism, rather do they ‘chop’ the human body into separate systems of organs and each one of those is treated by a narrow specialist which thus does not allow to form a comprehensive picture of a particular patient’s life and explain the symptoms within that context.

Now, if we take a helicopter view, we can say that development, mentalization, language acquisition and somatic disorders are not separate processes. They are all tightly linked with each other and this is rather an artificial splitting for the ease of conceptualization, research and observation but within a lifetime of an ordinary person they coexist and run in parallel. Schultz-Venrath (2024), a modern German psychoanalyst, attempts at unifying all these aspects together with the only exception of language. We find this approach very helpful for working with psychosomatic patients in psychoanalytic practice. In our article we are attempting to perform the same unification but adding on language acquisition. We assume that intersubjectivity is the best conceptual framework for integrated understanding of all these processes and at the same time it can be used as basis for describing transference-countertransference interactions. Recent theoretical developments underscore the importance of embodied simulation within the intersubjective communication (Gallese, 2007). This could be a potential scientific explanation for the transference-countertransference interactions observed in clinical practice of psychoanalysis and a promising direction for further profound grounding of psychoanalysis in the field of life sciences.

In this paper we attempt to focus on the intersubjective aspect of therapeutic interaction, on communication information through symptoms and on language as the main clinical tool in psychoanalytic psychotherapy, and on mentalization failure manifesting itself in an embodied countertransference reaction of a therapist. We are addressing the following questions in the article: why is body no less important in the intersubjective communication in psychoanalytic psychotherapy than language and how can language be applied as a clinical instrument for the treatment of bodily symptoms? What are the neuroscientific underpinnings of this communication and language application?

Talking about methodological aspects of this paper, we should emphasize that this is neither an exhaustive literature review, nor a meta-analysis or a randomized controlled clinical trial. This is a theoretical paper aiming at interdisciplinary integration. Our theoretical framework is based on extensive literature analysis and phenomenological clinical observations. Due to space limitations, we are not speaking about affective neuroscience and somatic therapies. We provide two small contrasting vignettes as illustrations of our conceptual assumptions. Finally, we discuss implications for therapeutic practice and training of future therapists and point out directions for potential further scientific research in the field of embodiment and intersubjective communication.

## 2 A psychoanalytic approach to the disturbance between mind and body

Over time, various psychoanalytic theories and orientations have reached a mutual agreement that the interaction between body and mind begins in the early stages of childhood when the infant depends on the caregiver for both physical and emotional needs (Winnicott, 1990; Beebe and Lachmann, 1994). During this early period, the child and maternal care form a unified body, and adequate caregiving requires the presence of a ‘*good enough*’ mother, as described by Winnicott (1975). This ‘*good enough*’ care creates a secure environment, essential for fostering a harmonious connection between body and mind. However, when the caregiver is unable to meet the infant’s needs or behaves inconsistently in response to the child’s demands, the continuity of being is disrupted.

As a defense against overwhelming affect, mechanisms such as dissociation and isolation are activated, potentially leading to a disruption between body and mind. In such cases, areas of the body may be perceived as dead, too tense, or deprived of feeling of life. As a consequence, the memory of this insufficient caregiving is blindly inscribed in the individual’s narrative as overwhelming affective experiences, whose traces remain at the somatic level. Sebastian Leikert (2021) calls them encapsulated body engrams, which sometimes can be felt even like a foreign body within one’s physical self. These engrams represent certain areas of psychical life where there is no connection between the somatic and symbolic level. But at the same time, the somatic level carries the meaning in a different form or in a different ‘language.’ Leikert (2021) describes it in the following way: ‘This bodily configuration carries an encoded meaning. This meaning, however, is not encrypted in words or images but in bodily tensions, which consist of petrified traces of the bodily resonance to catastrophic states’ (Leikert, 2021, p. 676). This ‘bodily resonance’ consists of sensory states through which the individual constructs and internalizes, early in life, the experience of good or bad caregiving.

In early infancy, the external and internal worlds are undifferentiated, and maternal care facilitates the development of a perceptual surface – referred to as the protective shield by Masud Khan (1963), or the psychic skin (or le *moi-peau* in French) in the terms of Didier Anzieu (1985). Through this process, the mother functions as an extension of the infant’s self, helping to bind excitations and providing containment for affective states. This early regulatory function of the mother is fundamental to what Freud termed the secondary process, which enables the transformation of drive energy into thought processes. This can be seen as the primordial mentalization process (Bateman and Fonagy, 2016).

Cumulative traumas might be considered breaches that disturb the mother’s role when she is, for some reason, unable to bind excessive excitation in the psyche of the child—being either nonresponsive or inadequately responsive to the child’s needs. According to Jean-Benjamin Stora (2007, 2015, 2023), who summarizes the economic viewpoint of the mentalization process based on Freudian ideas, the excitation in the psyche can be discharged via *motor activity*, somatic conversion into bodily symptoms frequently observed in hysterical patients, anxiety attacks at the psychical level, and finally, through being taken up by an external figure, which acts as a protective shield for the soma and psyche. The protective shield helps transform drive energy into thought processes (i.e., turning primary process into secondary process) and serves a dual function: it regulates internal drive excitations while also mediating interactions with the external world. When this protective shield is impaired, psychical functioning becomes significantly impoverished, resulting in somatic symptoms and a very concrete, operative way of thinking that lacks symbolization.

Freud (1923) famously stated, ‘the ego is first and foremost a bodily ego*’* (p. 26), highlighting the foundational role of the body in the development of self-experience. In summary, traumatic emotional needs that cannot be mentalized remain trapped within the body, manifesting as bodily symptoms, while cognitive development may accelerate prematurely. This imbalance contributes to distorted body representations at the psychic level, ultimately leading to precarious and fractured self-images in later stages of development.

At the early developmental stages, perceptions, emotional reactions, and actions are tightly linked with one another. This is normal due to the way the corresponding areas of the brain mature and acquire function. Prior to the development of mentalization, a child uses hallucinations as a primordial thinking process. Subsequently, as perception and motility advance further, the child becomes impulsive—emotions triggered by perception are immediately transformed into actions. For example, a one-year-old child looking at his mother recognizes her face (perception), immediately feels an affectionate reaction (emotion), and stretches out his hands to embrace her (action). As other areas of the brain develop—mainly the associative cortex—the child becomes more capable of thinking and using fantasy, i.e., of controlling hallucinations and therefore separating, in time and space, the three functions: perception, affective reaction, and action.

This is the moment when the transitional space emerges, and true symbols (by *symbol* we mean a freestanding, meaningful multisensory image linked with affective experience but unlinked from concrete action) can be formed within this space. This process runs parallel to, and is mediated by, language acquisition. At this point, more abstract semantic categories begin to emerge, representing one of the foundations of language development and use.

**Figure 1:**
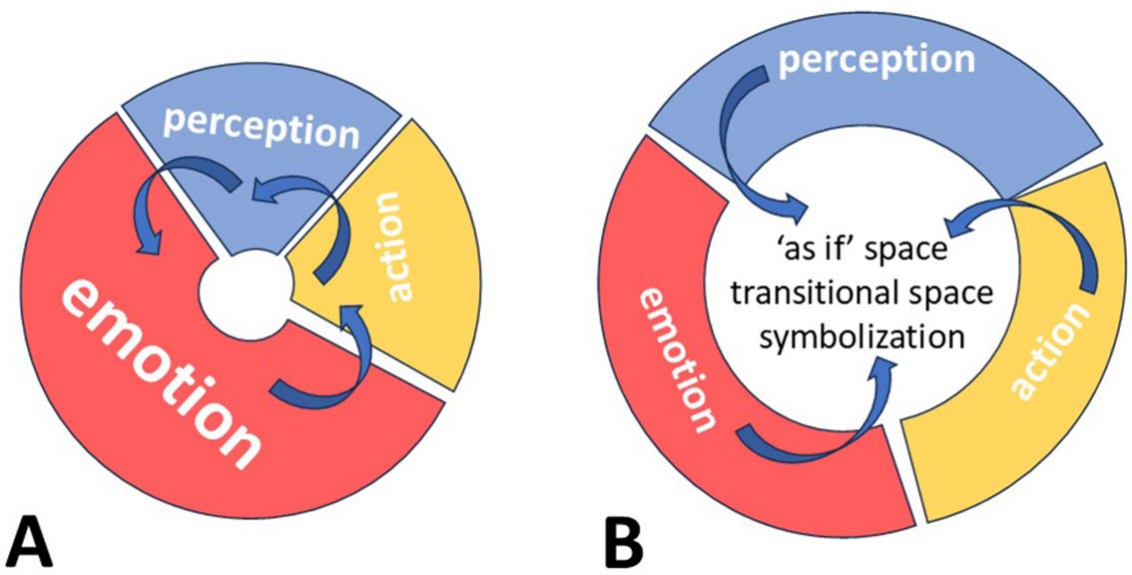
Emergence of transitional space within a psychic apparatus in development. It is a simplified abstract schema depicting the emergence of transitional space within a normal developing psychic apparatus which is shaped by several processes. Red segment of the diagram represents affective input; blue segment represents perceptional input (from the body and external world); yellow segment represents action input (in form of concrete or imaginary movement); a white hole in the center of each diagram represents the internal space for representation of the external reality necessary for subsequent development of thinking and self-reflection. Blue arrows indicate interactions between these processes. **(A)** Shows psychic apparatus at early stages of development with very limited internal space for thinking and representation. Here affective reactions prevail. Perception of external stimuli causes strong emotional response which is very quickly discharged in a concrete action. Here primary process dominates; **(B)** shows psychic apparatus at advanced stage of development, in which perception, emotional reactions and action are separated from each other by increased internal transitional space facilitating representation and thinking. Emotional reactions are better regulated and thus occupy a smaller segment. At this stage all three processes provide inputs for imagination, phantasy and ‘as if’ actions, which sustain symbolization. Here secondary process overrides the primary process.

These two developmental stages are depicted in the figure below (Figure 1). The early stage of development is schematically shown on the left, and the emergence of transitional space is shown on the right. In cases of physical or psychological trauma, or severe personality disorders, the transitional space may constrict—often regressing to an early developmental stage, a process psychoanalysis calls regression. Patients can then exhibit extremely concrete behavior, with no temporal gap between perception, emotional reaction, and action, as if no internal reflective space exists. Rather than thoughtful contemplation, they enact past traumatic experiences—or what psychoanalysis terms unconscious fantasies (Freud, 1908; Klein, 1932, 1948)

## 3 Symbolization and representation in the psychic space

Although development unfolds as a continuous flow, we will try to emphasize that the development of symbolization can also be understood and described in distinct stages. At first, the transitional space is formed which is indispensable for fantasizing, for imagination. Secondly, in this psychical space external objects can be represented as images or object-representations (or ‘*Sachvorstellungen’* in German). This is the simplest way of fantasizing and thinking because it’s still close to the concrete perception of the external objects. But as this process advances further, images are re-represented in internal and external experience through words. So, and thirdly external objects turn into words or word-representations (‘*Wortvorstellungen’* in German) (Freud, 1915).

Transitional space allows imagination and creativity to take precedence over reactivity. It is a place that permits thinking about feeling, as long as the child is not overwhelmed by external stimuli requiring forms of abreaction or bodily discharge, nor attempts to completely isolate itself from stimuli through the mind–body split.

Through this process, spoken language evolves into a unifying function of the mature psyche—integrating perception, memory, and emotion, and enabling intentional planning and action in the external world. As Winnicott noted, ‘an important basis for ego development lies in the individual’s communication with subjective phenomena, which alone gives the feeling of real’ (Winnicott, 1975, p. 188). Moreover, language structures our sense of time, weaving past, present, and future into a coherent continuity of the self (Bouizegarene et al., 2024, p. 18).

## 4 Language, emotional experience and somatization

Language serves many functions. Beyond communication, thinking, and planning—commonly described as its primary functions—it plays a crucial role in *binding* or representing emotional experience. When language is used *properly -* that is, when emotions, words, and actions flow together harmoniously—it can act as a *healing glue* for disruptions in the body–mind connection, a means of sharing subjective experiences, and a tool for translating bodily symptoms into words.

Language is gradually acquired through *good enough* emotional interactions between infant and caregiver, interactions that implicitly foster the formation of symbols (Winnicott, 1975, p. 98; Korkmaz et al., 2013). Once basic nurturing needs are met, the child begins to communicate desires and intentions across broader spatial and temporal domains. Children begin to adapt to the sounds and speech of those around them from the earliest moments of life, organizing their understanding of the world using the semantic meanings of other’s discourse long before they begin to speak their own words (Vivona, 2012). Moreover, symbols generate communication potentials that extend beyond their concrete referents, enabling children to share observations, ideas, and insights, and to engage in social activities such as gossip (Greenspan and Shanker, 2004). When children can use emotional signals to communicate, they are no longer overwhelmed by raw affect—raw affect that would otherwise collapse the transitional space and cause regression and symbolization failure (see previous section).

Sharing emotions is an important way to regulate them, and language mediates this sharing. As Green (2005) put it, ‘Language without affect is a dead language. And affect without language is uncommunicable. Language is situated between the cry and the silence’ (Green, 2005, p. 205). Linguistic meaning is anchored in bodily experience and sensorimotor processes (Gallese et al., 2007). Moreover, the body and bodily experience represent the necessary context for any kind of human experience, with language being, in fact, an embodied aptitude whose origins lie in empathic communication (McGilchrist, 2012). Thus, language can be described as either affect-bound or affect-unbound.

Affect-Bound (Embodied) Language bridges the individual’s internal affective world with external reality. Rooted in sensorimotor and emotional resonance, it relies on internal simulations of one’s own affective states and the intersubjective mirroring of others. Affect-bound language enables balanced emotional expression and processing. Its foundations lie in the body and the realm of experience and corresponds closely to what we term *embodied language*.

In contrast, affect-unbound language is disconnected from the speaker’s affective universe and emotional experience. In fact, it is often used defensively to avoid conscious engagement with affect. In discourse, it often manifests as a denotative discourse lacking authentic emotional involvement or disconnected from the speaker’s affective world. Affect-unbound language frequently correlates with excessive somatization, in which unprocessed affect finds its outlet in bodily symptoms that remain difficult to understand or interpret (Schultz-Venrath, 2024, pp. 63–64).

We think that therapy can restore the bridge between felt experience and symbolic representation by fostering affect-bound language, thus reducing the need for somatic expression of unmentalized emotion.

### 4.1 How do we learn to speak the language, and how can it be used in therapy?

Much research and numerous theories address language acquisition. Here, we focus on two landmark observational studies relevant to our discussion: Michael Halliday’s longitudinal study of his son Nigel (Halliday, 2004) and Kornei Chukovsky’s pedagogical observations of children in early childhood (Chukovsky, 1990). Halliday (2004) observed his son Nigel from 9 months to 2.5 years and Chukovsky registered his observations of different children from 2 to 5 years old with whom he worked as a pedagogue. These two works cover the developmental period of children when their main activity is play and when they master how to speak the language. Halliday (2004) explains how language is acquired as a tool for various functions, of which the most important is abstract thinking whereas Chukovsky describes how this acquired tool can be used for playful behavior which develops creativity and deepens the understanding of the complexity of social interactions and cultural phenomena.

### 4.2 Halliday’s three phases of early language development

Halliday (2004) divided this period between 9 months and 2,5 years in three phases from the sociolinguistic position. Phase I (approx. 9–17 months) is the child’s initial functional-linguistic system; Phase II (approx. 17–18 months) is the transition from this initial system to the adult language; Phase III (approx. 18–24 months) is learning of the adult language. At Phase I there is no grammar, no level of linguistic forms, a child has primitive symbols expressed in primitive phonetic expressions but there are no lexical forms. It has nothing to do with a particular language, sounds are spontaneous and are mostly the imitations of natural sounds and the sounds produced by the caregivers. Notwithstanding all the limitations, a child can still satisfy his own needs, can manipulate the behavior of other people, establish contact with caregivers and express his own individuality. At Phase II grammar and vocabulary emerge and a child becomes capable of expressing more specific choices. At this stage language acquires its cultural specificity (the English language, the German, the Chinese). Vocabulary helps adding new meanings within functional behavior and facilitates combination of functions. At this point a child becomes able to convey new information to the caregiver and categorize observed phenomena in a more complex way. A child at this stage is oriented at the communication with the other and starts using language for simple dialogues. At Phase III finally the child further develops the two main forms of meaning potential, the ideational one which helps representing experience, interpreting the internal and external reality (i.e., abstract thinking or symbolization) and the interpersonal one which is used for communication and social action.

Through these phases, the child progressively abstracts from immediate experience, culminating in the capacity for complex thought (Halliday, 2004).

### 4.3 Chukovsky on playful language creativity

Chukovsky (1990) further expands on the play-like behavior and its connection to the language. He states that each child needs to be an explorer of his native language through creativity. Children start with simple repetition of words, colocations and phrases but afterwards when they master the language in an emotional way they start creating neologism, i.e., new words, non-existent in the native language but depicting the sense of their experience, i.e., they are training the ideational potential of the language in their minds, the one acquired at Phase III according to Halliday (2004). Neologisms are new forms and they depict the playful aspect of this exercise. Neologisms are usually formed through mastering of endings, prefixes and suffixes, depicting the size of objects (big or small), relational aspects (mine, her, his, theirs) and the emotional value of the experience (joy, fear, sorrow). Chukovsky (1990) emphasizes that while studying the grammatic structure of language the child *creates* the internal rules or structure of the language by trying new things, outside of the conventional language rules which is a form of play. He emphasizes how creative children can be in the way they use words when they describe their emotional experience in stable relationships with adults with secure attachment, in a safe environment. Chukovsky (1990) says the following: ‘*The child’s giftedness in speech consists not only in the classification of endings, prefixes and suffixes, which he produces unnoticed in his two-year-old mind, but also in the guesswork with which he chooses the necessary pattern for imitation when creating a new word. Imitation itself is here a creative act*’ (Chukovsky, 1990, p. 85, translated from Russian by Elena Markova). Children tend to ‘animate’ inanimate things by using corresponding suffixes, for them everything around is animated. And also, they frequently make nouns out of verbs and verbs out of nouns, trying out new word forms, like avant-garde poets. Chukovsky (1990) says further about other children who grew up in narcissistic conditions with emotionally distant parents: ‘In the olden days, I happened to meet children who, for various reasons (mostly at the whim of wealthy parents), were imposed from infancy on the vocabulary and structure of a foreign language, most often French. These unfortunate children, cut off from the elements of their mother tongue from the very beginning, knew neither their own language nor that of others. Their speech in both cases was equally anemic, bloodless, dead - precisely because they were deprived of the opportunity to master it creatively between the ages of 2 and 5’ (Chukovsky, 1990, pp. 84–85, translated from Russian by Elena Markova). He calls this language gift in children at this particular age *unconscious artistry*. Finally, he says that this creativity in language construction observed in children at the age of 2–5 disappears completely by the age of 8 when they have fully mastered the native language.

## 5 Embodied simulation and intersubjectivity in psychotherapy

Since we have just described the importance of symbolization and language acquisition, we should now go back to the body perception and to the role that it’s playing in intersubjective communication because this topic seems to be frequently overlooked in classical psychoanalysis though nonverbal communication plays one of the key roles in our psychotherapeutic practice and we would like to bridge the gap between body and mind and point in the direction of how we can restore the harmonious continuum between the body and symbolic representation in the mind in our patients.

When we are talking about human beings especially in the times of active development of artificial intelligence it is important to emphasize that we are not just large language models or just genetic information assembled in vacuum. We are embodied organisms and our subjectivity and the sense of self are tightly linked to this simple fact. Our body is an important instrument for communication with the external reality including other human beings since we live within a social environment. Gallese and colleagues suggested a theory of embodied simulation Gallese (2007) based on the discovery of the system of mirror neurons that help us instantly perceive information about the goal directed behavior and emotional state of the other. D’Adamo et al. (2024) postulate that intersubjectivity can be called intercorporeality which is described as mutual resonance of intentionally meaningful sensorimotor behaviors.

This implicit nature of intersubjective communication is vividly observed in the phenomenon of contagious behavior when individuals spontaneously mimic behaviors and emotional expressions of the others. This is the evolutional acquisition and a basis of effective social engagement. Motor resonance between two people can lead to emotional contagion when the internal state is instantly transmitted to the other. But we all differ in susceptibility to the motor and emotional states of the other due to the flexibility of the distinction between self and other. As D’Adamo et al. (2024) point out, this is based on the brain’s ability to merge synchronous inputs from different sensory modalities thus creating the unified perception of self and the environment which is called multisensory integration characterized by so called temporal binding window (TBW). This is the temporal tolerance window within which two stimuli are integrated and it varies across population but can be flexibly changed with training as D’Adamo et al. (2024) showed in the recent study. The wider the TBW the more prone a person will be to contagious behavior or we can call it increased capacity for embodied simulation. On the other hand, this refers to the stability of the perception of self and of the me-not me boundary. The wider the TBW the less stable the perception of self and the more overwhelming the perception of the other might thus be. The ability to adjust TBW width should remain flexible and reversable depending on the intersubjective context which is to some extent an important social skill and on the other hand is defined genetically. D’Adamo et al. (2024) shows that it can be exercised and it does not take much time though this flexibility can be limited due to certain psychopathology like autism, schizophrenia or BPD.

This contagious behavior is crucially important in psychotherapy. Thomas Fuchs (2016) suggested a concept of primary or pre-reflective intersubjectivity based on embodied affectivity. He indicated that two individuals in dyadic relationships communicate through bodily resonance and connect with each other in circular interactions (Fuchs, 2016). Later on, this idea has been described in terms of simultaneous brain activity of patients and therapists through inter-brain synchrony. Coupling of brain activity within a therapeutic setting lies at the heart of connectedness between the two persons. This mechanism of inter-brain synchrony and plasticity was described by Sened et al. (2022) In fact, the brain (and the body) of one person forms a sort of a common *neuronal network* with the brain (and the body) of the other person through multiple circles of repeated activation in close succession immediately one after another. So, it means that when we are engaged with the other person in some activity involving high inter-brain synchrony it increases the ability to synchronize and this capacity or trained skill can afterwards be applied in other intersubjective communications. This is exactly what is being trained session after session in the psychotherapeutic setting and is basically the essence of the psychotherapeutic process in itself. It was demonstrated that experienced psychotherapists are much quicker in synchronization with their patients while in psychopathologies like aforementioned BPD and schizophrenia this skill appears to be very limited leading to huge problems in social communication. It has been described in clinical practice that not only severe congenital psychopathologies but also transitional pathological conditions like melancholia or clinical depression can result in desynchronization of an individual with the environment and society thus causing a feeling of falling out of time and space. Psychotherapy is thus needed as a tool for ‘resynchronization’ with the time flow and the society (Fuchs, 2005). Generally speaking, all psychiatric pathologies can be characterized by impaired synchronization with other people manifesting itself as changed sense of other people or loss of access to them which can be experienced as an existential threat (Ratcliffe, 2008; Gallese, 2006).

### 5.1 Embodied cognition and language

The recent trend of viewing the brain and mind through computational lenses inevitably leads us to consider that scientists working to understand the mind might apply the same model of functional architecture to “artificial brains,” namely Large Language Models.

Lately, more and more people have begun engaging in conversations with these artificial models for various purposes—some of which relate to mental health, such as alleviating loneliness, seeking therapeutic advice, and many other reasons (Rousmaniere et al., 2025). In fact, many people see LLMs as a fairly good alternative to psychotherapy with a real person, especially since it is always available and costs almost nothing (Bucher et al., 2025). But can Large Language Models truly replace a real therapist, given that we are speaking of a process in which intersubjectivity unfolds in relation to an artificial entity that possesses neither a body nor the capacity for bodily resonance?

The accounts of artificial intelligence express a reality detached from the body—one that seems to be composed of fragments combined much like words arranged through syntax—and which differs in many ways from the accounts of a subjective experience. Large Language Models construct and understand things through imitation or by assembling multiple elements together in order to provide a form of coherence to what is said, much like confabulations (Wiest and Turnbull, 2025). Confabulations are grounded in anything but a relationship with reality, and this makes them, no matter how concrete or seductive they may appear, devoid of reliability. Reality testing requires, above all, not only the ability to generate viable predictions but also the capacity to validate or correct these inferences through the senses. However, artificial intelligence is incapable of bodily resonance. Its reality is represented, not re-presented, because re-presentation involves not only imitation but also the existence of a body capable of subjectively experiencing and expressing its own or another’s emotional world. Thus, when asked about its own experience or about the experience of another person, any LLMs manages to confabulate one that represents, in fact, only a conceptual and metaphorical language—yet a disembodied language lacking any affective connection, whether it is about a connection with its own emotions (which, in fact, do not exist) or with the emotions of the other (which it cannot feel and to which it cannot have access in the absence of language). Therefore, the intersubjectivity of Large Language Models (LLMs) seems to be expressed rather as “intrasubjectivity,” where their accounts can be considered an extension of the human mind that reproduces a disembodied model of thinking. It is similar to inner speech that imitates ways of being or feeling in multiple forms, yet without an authentic affective relational experience.

However, human-to-human interaction, and implicitly body-to-body interaction, involves much more than a cognitive or language model. Every interpersonal interaction involves the sharing of sensory and emotional states, and intercorporeality represents the primary source of our knowledge about others (Gallese and Cuccio, 2015). And this is crucial in the psychotherapy process. People who seek help through psychotherapy are individuals whose past has been, and continues to be, strongly encoded in their sensory experience. Thus, embodied language and cognition provide us with diverse ways of understanding others, which is particularly important in therapeutic practice. Gallese (2003) refers to this mechanism as “experiential understanding” (p. 3), where the sharing of intentions, emotions, and the meanings of actions with others creates a meaningful interpersonal space. This space is formed from birth through the dyadic interactions between mother and infant and is later unconsciously reactivated in other types of interpersonal relationships, not only in therapeutic ones. The very phenomenon of countertransference is based on the therapist’s ability to enter such resonance—something that is impossible for Large Language Models when they are used to provide therapeutic advice to users. In fact, Gallese (2009) states that “the body is the main source of meaning, because it not only structures the experiential aspects of interpersonal relations, but also their linguistic representations. This proposal can stimulate a new form of dialogue between neuroscience and psychoanalysis, based on the common goal of grounding the analysis of human experience on a multilevel and multidisciplinary approach, likely the sole capable of succeeding in the fascinating enterprise of understanding who we really are” (Gallese, 2009, p. 533).

Thus, sensory information processed through the senses, which are strongly anchored in the body and somatic experience, as well as the motor dimensions of interpersonal experiences, play a very important role in the production and understanding of language (Gallese and Cuccio, 2015). In fact, embodied simulation may play a crucial role both in providing meaning and in understanding language from interpersonal experiences, which is even more significant in the phenomenon of countertransference. As therapists, we understand and experience our patient’s internal worlds through our own bodily experience, and the enactment of this bodily simulation process in understanding the meaning of symptoms—and implicitly of language—suggests that the symbolic and bodily dimensions coexist in the construction of linguistic meaning (Gallese and Cuccio, 2015). We believe that the symbolic dimension, together with linguistic meaning, helps construct meanings associated with the bodily simulation of the other’s experience and its verbalization. In line with this, we will further illustrate the above discussion through a brief clinical vignette.

### 5.2 A small clinical vignette

Let us now have a look at how we can use the concepts of embodied simulation, inter-brain synchrony and contagious intersubjectivity to explain some striking clinical phenomena that we sometimes encounter in our office. Here is a small clinical vignette presented by the second author of the present study which illustrates these processes within the intersubjective field between a patient and a psychotherapist.

I was working on ZOOM with a patient in his mid-forties who had a severe narcissistic personality disorder. He was a chronic drug abuser with various somatic symptoms but his gastrointestinal tract was the most affected system. He had a gastric ulcer, ulcerative colitis and he also had a functional dyspepsia which resulted in bile vomiting. All these symptoms were reactions to separation from an attachment figure or to frustrations of immediate satisfaction of his desire to be cared for. He was extremely needy, breaking all the rules. At the beginning of our treatment, he sometimes came to sessions high on drugs and had several suicidal attempts to make me feel anxious about him and possess all my attention.

His mother was a very beautiful woman; a model and she never breastfed him because she wanted to preserve her breasts for model business. He was abused by his mother physically and emotionally as a child and he interrupted all communications with her upon turning 16. He was sometimes mad with rage towards his mother though he had not seen her for many years. At some sessions he confessed that he wanted to murder her in the most perverted and bloodiest way and at subsequent sessions he said that he missed her still.

He usually said that he feels himself a hungry man without a mouth. He felt the same way in close relationships. He was desperately trying to connect to the other person and each time failing to do it. In my countertransference with this patient, I felt a strong tension and irritation. But even more striking to me was the fact that he several times attempted to terminate our therapy and each time I developed a very painful bloody hemorrhoids which I had never ever had before this case. We worked for 2 years and during the termination phase of our treatment he had a bleeding ulcer perforation and had to be operated and at the same time I again developed a severe bleeding hemorrhoid.

In fact, this is a vivid illustration of a very close connection between body, symbolization and countertransference reactions. We can see that lack of symbolization on behalf of both participants (the patient as well as the therapist) of this interpersonal communication due to enormous strength of affective states led to a very concrete embodiment of countertransference reactions, resembling a ‘copy-paste’ function in a digital text document. Subjective narrative was communicated from one person to another not at the level of spoken words or language but rather at the level of the body and its symptoms which is actually an example of embodied simulation.

## 6 Discussion

Since in this article we set out to explore the connection between bodily and mental processes, we would like to further highlight a few aspects regarding the synchronization between patient and therapist—both at the mental level, through language, and at the brain–mind and bodily level, through mirror neurons.

As we know even from our lived experience some phrases and words can be highly *contagious* and can be easily passed over from one person to another within the intersubjective space of close personal communication. But emotionally bound words are usually linked to unique personal experiences or better to say memory traces. On the other hand, language is also an important part of a cultural context. Thus, language becomes a certain *glue* as we mentioned above between internal lived emotional embodied experience of a person and the external environmental context within which a person was born and grew up. A therapist has to study the individualized language of each patient, his internal vocabulary and cultural environment. Through this study of the other person’s language therapists synchronize with patients. This happens at a mental level within the transitional space which is an interpersonal environment of safe attachment potentiating playful activities and active investigation of the other person’s mind.

On the other hand, the same synchronization happens between two people at a bodily level bypassing the mind conscious awareness.

The *talking cure* is one of the psychotherapeutic tools which helps us to unite these various types of synchronizations and convert bodily transferred information into text and integrate it into the patient’s individual narrative.

### 6.1 Large Language Models versus embodied simulation

It is interesting and challenging to compare interaction of a humans with LLMs and the interpersonal communication between the therapist and the patient in presented clinical vignette. In both dyadic systems spoken language is used for communication and on the surface, it might look the same but in case of LLMs these are empty words without the true meaning because AI has no body and therefore no lived experience. LLMs only imitates the conversation, acts as if it is a living organism though it has no embodiment. In this case language is used to imitate the real-life situation. It has no true meaning for the artificial system itself compared to the embodied human being behind the screen. Whereas in case of communication between the therapist and the patient described in clinical vignette the situation is absolutely different or even contrary, some part of information is left unspoken but it is still present in the interpersonal setting and is transferred directly through the body though all communication was conducted in the virtual reality, via ZOOM and it was a ‘talking cure’. This depicts how much of essential information is transferred through the body between the two people even when both of them are partially ‘hidden’ behind the screen in ZOOM videoconferences. This bodily symptom disappeared once the therapist was able to mentalize it, i.e., convert into explicit narrative. In this narrative all words are meaningful because they are connected with development and lived experience entailing existential threat. Therefore, we can say that the body knows more than does the mind. Mentalization is a much slower process, it takes time and it’s a skill that should be acquired during development and ‘taught’ for those patients that have a limited capacity for that.

Summarizing the whole issue concerning LLMs, we can say that trying new things and expanding your lived experience within a close interpersonal communication with a living, embodied Other, in the safety of a transitional space of psychotherapy that facilitates the development of your own self-narrative, is qualitatively different from expanding your understanding of life through reading books or watching digital materials—and even less so through conversations with LLMs. This is what Bion called learning from experience and it also refers to the famous thought experiment with blind Mary described by William James. The embodied experience fundamentally changes your understanding of a certain phenomenon. Once experienced it cannot be completely forgotten anymore.

### 6.2 The importance of countertransference

Now as we have emphasized the importance of a body in communication with patients then we can pose a further question. How does this embodied simulation happen? How can we explain it from two different perspectives, a psychoanalytical and a neuroscientific?

We postulate that such pathological resonance in the body of the therapist is the result of affect amplification due to projective identification and a strong countertransference reaction. It is worth mentioning that differentiation between projective identification and a deeper underlying countertransference reaction is very important in psychoanalytic psychotherapy and is frequently overlooked. These two phenomena can easily be confused as they tend to merge together in the psyche of the therapist if there is not enough depth of self-awareness. In this particular case there was a problem on behalf of the therapist as she had the same rageful attitude towards her own mother due to certain experiences of her early childhood and this deep traumatic trace had not been worked through by the time of therapy with this patient. This blind spot produced an unconscious collusion of the therapist and the patient based on the idea of a sadistic murder of an abusive mother. This collusion gives sort of a green light to projective identification on behalf of the patient. The role of a bad mother is then attributed to the therapist which is projective identification and this agreement to accept the role and the masochistic submission to a sadist is the deeper countertransference reaction on behalf of the therapist. We can call it an embodiment of the unconscious phantasy or a pathological form of brain–body synchronization in the intersubjective field. We can also hypothesize that the therapist has an increased capacity for synchronization and her temporal binding window (TBW) is too wide.

Another possible, speculative translation of psychoanalytic terms ‘countertransference reaction’ and ‘projective identification’ into neurobiological concepts can be the mechanism known as primary and secondary intersubjectivity described by Gallagher (2005). These are very early non-mentalistic mechanisms of communication which are pre-theoretical knowledge about how people behave in particular contexts based on embodied practices with others. He claims this mechanism is congenital and is present even prior to embodied simulation which is a developmental acquisition.

### 6.3 Body mentalization through embodied simulation and somatic narration

Another important question for clinicians is how can we help poorly metalized patients like the ones with BPD and NPD relive their various somatic symptoms? It can be hard to work with such patients using the talking cure from the very beginning, since they tend to use language defensively and lack symbolization to avoid thinking about some unpleasant experiences associated with painful affective states and problems that look unsolvable to them. This is what Bateman and Fonagy (2004) call `pseudomentalization` which on the surface presents itself as a coherent and smooth narrative but in fact it lacks the true emotional content on behalf of the patient. These are lifeless cognitive constructs detached from the embodied experience of the individual. In these cases, symbolic function should be restored and pseudomentalization should be turned into a genuine mentalization process which facilitates the reconnection between the body and the mind.

In order to relive the symptoms we need to help patients create their own ‘somatic narration’ (Leikert, 2023). But before explicit expression of the unconscious somatic content in the form of verbal language can be achieved the embodied preverbal level of interpersonal communication should be used by the therapist to facilitate development of mentalization function in the patient. And this process relies fully on the embodied simulation through interbrain synchronization and countertransference analysis done on behalf of the therapist. Bateman and Fonagy (2016) calls it ‘shared affect’ between patient and therapist (p. 255).

Further technical aspects of such mentalization-enhancing therapy in patients with BPD can be found elsewhere (Schultz-Venrath, 2024).

### 6.4 Implications for clinical practice, training of psychotherapists and further research

We claim that more attention should be paid to nonverbal aspects of communication and bodily synchronization within therapeutic process and neurobiological underpinnings of these processes should be taught in clinical training for psychoanalytic psychotherapists for better understanding of these mechanisms. Video recordings of patient-therapist sessions can be used for the analysis of embodied simulation within the therapeutic process though it’s not a common practice in contemporary psychoanalytic psychotherapy. Therapists should learn how to translate implicit information into a narrative and how to help patients mentalize bodily symptoms based on transference analysis.

As now we are facing the advances of current AI technologies and lots of people are using LLMs as an auxiliary therapist (especially patients suffering from autism spectrum disorders, schizoid and schizotypal personality disorders) it should be clarified for them that it is a simulation of human contact rather than a real one. Moreover, there is a warring tendency for people with acute mental distress, namely psychosis, mania and suicidal ideation, to use LLMs for help rather than apply for professional psychiatric support (O'Dowd, 2025). Potential social stigma could be the underlying reason for such kind of biased choice of helping strategies. But LLMs has no embodiment and therefore is not a living human being capable of understanding their sufferings and too frequent application of such ‘mental support’ can result in addiction, termination of normal therapy with a trained analyst, formation of unrealistic beliefs and expectations, sometimes leading to lethal consequences like real suicide attempts^1^. We think that discussing application of AI technologies with all the dangers it entails at the beginning of treatment should be part of contracting and setting formation in current reality.

Clinical observations of inter-body synchronization suggest further questions for research. Countertransference has been described predominantly as a psychical phenomenon but since it involves the body of a therapist it could be studied in more details for better understanding of the interpersonal communication. Since therapists use their whole personalities (i.e., body and psyche) for treatment of patients it is obvious that body resonance is a very important tool for understanding the other given that one knows his own structure not only at a psychical level but also at the level of embodied reactions. Countertransference, especially through embodied simulation is not an obstacle in psychotherapy. On the contrary, it’s a rich source of information about the unconscious interpersonal communication and it can also be expressed at a bodily level if a therapist resonates with a patient with his own unsymbolized traumatic memory traces as described in the presented vignette.

Other daring and speculative questions can be posed about potential ‘transfer’ of bodily symptoms. The idea is that if behavior can be contagious through inter-body resonance and synchronization, can severe diseases be possibly ‘contagious’ in the same way or maybe through a different mechanism? If a body posture of one person can mirror a body posture of the other person can the same apply to the internal organ systems or not? Or maybe there could be a cross-talk or so to say transference-countertransference interaction between two individuals at the level of organs? It is also important to emphasize at this point that we are not inferring any physical hypothesis for such kind of transfer or any literal concrete causality, we are rather using the words ‘transfer’ and ‘contagious’ more in a symbolic sense, as a clinical metaphor. Though, at the same time, we suggest paying more attention to the engagement of the immune system in interpersonal synchronization which might be of particular interest for further research. For example, it has been described in 2010 that mere visual perception of disease-connoting cues can promotes an aggressive immune response attenuated by increased levels of proinflammatory cytokine interleukin-6 (IL-6) (Schaller et al., 2010). Another recent study published in 2025 described activation of innate lymphoid cells as part of anticipatory neural response to potential infection in contact with infected people (Trabanelli et al., 2025). These are just a few illustrations, obviously further experimental research is needed for the description of interpersonal reactions at a deeper level, involving not only neuronal networks but other organ systems as well.

Finally, it would be of interest to study long-term impact of utilization of personified AI assistants by patients first and foremost on their mental health, mostly its role (if any) in provoking psychotic episodes and suicidal ideation, and compare that with ordinary CBT, psychoanalytic psychotherapy and pharmacotherapy. Influence of personified AI assistants on bodily symptoms of psychosomatic patients is no less interesting for mental health specialists because for now the information is quite scares.

### 6.5 Limitations

It is essential to emphasize that this article has a number of important limitations. First of all, it’s not a systematic review or meta-analysis or fundamental research suggesting level A evidence. Our aim was to integrate different theoretical approaches, namely psychoanalytic theory, neuroscience and developmental psychology, to the problem observed first and foremost in our everyday clinical practice with patients, mainly that bodily symptoms are hard to deal with in the frame of so called ‘talking cure’. However, any scientific endeavor aimed at understanding and treating psychopathology that takes into account both patients’ subjective bodily experiences and the ways in which these experiences reverberate intersubjectively within the therapeutic relationship must be methodologically rigorous and responsible. Accordingly, longitudinal and controlled studies conducted on sufficiently large and representative samples are required in order to capture both the objective dimension and the patient’s subjective experience. Moreover, it is important to investigate the intersubjective mechanisms involved—such as nonverbal synchrony, affective co-regulation, and shared bodily attention—and to test interventions that integrate bodily work (embodiment) within talking therapies, evaluated through randomized controlled trials wherever possible.

How can bodily symptoms be understood, interpreted and translated into spoken language? That’s a question that psychoanalysis has been addressing since the early 1920s. However, it might be of interest to perform a systematic analysis of data published on therapist’s countertransference reactions associated with patient’s symptoms. This might give a broader picture of the scale of this phenomenon, search for correlations with other factors (like genetic markers, history of mental health problems in therapists, etc. etc.). Another possible option for further research could be a systematic analysis of special lexica utilization in different subpopulations of patients which could be used for psychotherapeutic treatment because changing a subjective narration can change general condition of a person in a top-down manner and might even affect somatic symptoms. Secondly, some ideas that we bring up and discuss in this article might sound speculative, abstract or too general, like for example, countertransference reactions and embodiment of unconscious phantasies illustrated in the clinical vignette. It should be clarified, that this vignette is rather an illustration of countertransference somatization which might be of interest for further scientific investigation. It should not be overgeneralized and interpreted as sufficient evidence. This is rather a phenomenological observation. At the same time, it would be interesting for psychoanalysts to describe more of such observations of embodied countertransference reactions in papers and not at the level of just a somatic description but rather with underlying phantasies on both sides, the therapist and the patient. This might be difficult due to personal disclosure and confidentiality issues but very helpful for deeper understanding of the underlying mechanism of this clinical phenomenon. Thirdly, thoughts about LLM utilization might be too short and tangential. Our idea was rather to emphasize the contrast of intersubjective communication between two living embodied beings versus communication between an AI system and a human being. Nonetheless, it is very important to carry out an international prospective multicenter surveillance study with different cohorts of mental health patients (e.g., patients with major depression, with bipolar disorder, with schizophrenia, with somatic symptoms disorder, etc.) utilizing LLMs alongside with psychotherapy or quitting therapy for subsequent LLM ‘treatment’ whenever this information becomes available to healthcare specialists, with a particular focus on long-term consequences including psychotic episodes and suicide ideation. This could help carefully develop clinical guidelines in the future indicating limitations and possible threats of AI utilization for patients with various mental health problems.

## 7 Conclusion

Body is a valuable source of nonverbal information about a patient which is conveyed through synchronization or embodied simulation to a therapist within the intersubjective communication and language is used in this process for mentalization or symbolization and restoration of the body–mind connection through creation of a coherent somatic narration. We want to draw attention of both, researchers and clinical practitioners to this issue once again because there are still a lot of open questions to be addressed which we tried to indicate here in the discussion section. And another important issue is cautious application of modern technology, since ubiquitous AI utilization by patients is becoming more and more apparent and significant in the field of mental health in the recent months with some alarmists stating that AI will soon substitute psychotherapists ‘of flesh and blood’ and dominate psychotherapy (Frances, 2025) which we claim sounds like a truly disastrous perspective for healthcare due to reasons that we attempted to outline in this paper.

## Data Availability

The raw data supporting the conclusions of this article will be made available by the authors, without undue reservation.

## Footnotes

1
https://www.theguardian.com/technology/2025/apr/15/she-helps-cheer-me-up-the-people-forming-relationships-with-ai-chatbots?CMP=Share_iOSApp_Other
